# Heavy Metals in Water Percolating Through Soil Fertilized with Biodegradable Waste Materials

**DOI:** 10.1007/s11270-016-3147-x

**Published:** 2016-11-22

**Authors:** Jadwiga Wierzbowska, Stanisław Sienkiewicz, Sławomir Krzebietke, Teresa Bowszys

**Affiliations:** Faculty of Environmental Management and Agriculture, Department of Agricultural Chemistry and Environment Protection, University of Warmia and Mazury in Olsztyn, Oczapowskiego Street 8, 10-719 Olsztyn, Poland

**Keywords:** Trace elements, Composted, Sewage sludge, Municipal compost, Leachate, Leaching

## Abstract

The influence of manure and composts on the leaching of heavy metals from soil was evaluated in a model lysimeter experiment under controlled conditions. Soil samples were collected from experimental fields, from 0- to 90-cm layers retaining the layout of the soil profile layers, after the second crop rotation cycle with the following plant species: potatoes, spring barley, winter rapeseed, and winter wheat. During the field experiment, 20 t DM/ha of manure, municipal sewage sludge composted with straw (SSCS), composted sewage sludge (SSC), dried granular sewage sludge (DGSS), “Dano” compost made from non-segregated municipal waste (CMMW), and compost made from municipal green waste (CUGW) was applied, i.e., 10 t DM/ha per crop rotation cycle. The concentrations (μg/dm^3^) of heavy metals in the leachate were as follows: Cd (3.6–11.5) < Mn (4.8–15.4) < Cu (13.4–35.5) < Zn (27.5–48.0) < Cr (36.7–96.5) < Ni (24.4–165.8) < Pb (113.8–187.7). Soil fertilization with organic waste materials did not contaminate the percolating water with manganese or zinc, whereas the concentrations of the other metals increased to the levels characteristic of unsatisfactory water quality and poor water quality classes. The copper and nickel content of percolating water depended on the concentration of those metals introduced into the soil with organic waste materials. The concentrations of Cd in the leachate increased, whereas the concentrations of Cu and Ni decreased with increasing organic C content of organic fertilizers. The widening of the C/N ratio contributed to Mn leaching. The concentrations of Pb, Cr, and Mn in the percolating water were positively correlated with the organic C content of soil.

## Background

Sewage sludge produced in wastewater treatment plants should be disposed of, in accordance with the national Waste Management Act of 14 December 2012, for legal, esthetic, and practical reasons. Due to its high content of organic and biogenic compounds, sludge is a rich source of nutrients (nitrogen, phosphorus, magnesium, calcium, and sulfur) required for plant growth, and it can be used for agricultural purposes. Sludge contains many nutritious substances and organic matter that improve soil structure, which is why it can be used as organic fertilizer and for reclamation of degraded soil (Bień et al. 2011). However, the application of sewage sludge to land is permitted only if the concentrations of trace elements in the sludge and topsoil do not exceed the specified limits.

In Poland, similarly to other European countries, a major part of sewage sludge from municipal wastewater treatment plants is used in agriculture (GUS 2015). This is the simplest and cheapest method of permanent disposal (Sadecka et al. 2011), consistent with the principles of municipal sewage sludge management set forth in the National Waste Management Plan 2014, which outlines perspectives for 2015–2022, aimed at maximizing the recovery of biogenic elements from sewage sludge. In many cases, this is also the only option available for the use and disposal of municipal sewage sludge in small- and medium-sized wastewater treatment plants that produce sludge characterized by moderate or low levels of heavy metal contamination (Wilk and Gworek 2009; Wieczorek and Frączek 2013). Trace elements contained in organic waste materials used as fertilizers can be a source of nutrients for plants, but, when present in high concentrations, they can also pose a threat to both the plants and the soil-water environment (Xiang et al. 2000; Bowszys et. al. 2009a , b; Gawdzik 2012; Ciszewski and Grygar 2016) and, consequently, become incorporated into the food chain. Heavy metals found in sewage sludge can be dissolved, precipitated, co-precipitated with metal oxides, adsorbed, or associated with particles of biological residues (Alvarez et al. 2002). The rate of heavy metal leaching from soil is dependent on the pretreatment of sewage sludge (Milinovic et al. 2014), and on overgrown vegetation and the depth of groundwater (Lipińska et al. 2016). According to Fodor and Szabó (2004), the rate of transformation and immobilization of toxic pollutants in soil depends on the type of pollutant. Some heavy metals are mobile in soil (Cd, Zn, Pb, Cu), whereas others are rapidly bonded into insoluble forms (As, Hg, Cr). According to McLaren et al. (2005), the rate of metal movement down the soil profile is determined by the types of land use and heavy metals; Cd, Ni, and Zn were the most mobile in soil, Cu and Pb were less mobile, whereas Cr was the least mobile metal. These elements contained in sewage sludge were more mobile in forest soils than in pasture soils. Baran et al. (2011) analyzed the leaching of trace elements from mountainous meadow soils and found that metal concentrations were considerably higher in the leachate from soils with organic fertilization (sheep penning) than from treatments with mineral fertilization. The soil leachate contained the lowest concentration of nickel, higher concentrations of copper and manganese, and the highest concentration of iron. The lowest amounts of copper and nickel were leached from the NPK treatment, and the lowest quantities of manganese and iron were leached from the control treatment. Pirani et al. (2006), who analyzed trace element leaching from poultry litter-amended pastures, reported that reduced application rates of poultry litter did not rapidly decrease the amounts of As, Cd, Se, Cr, Mn, Fe, Ni, Cu, and Zn leached out to subsurface waters.

The aim of this study was to determine the effect of organic fertilizers on the leaching of trace elements from soil.

## Materials and Methods

The field experiment was established on Haplic Luvisol (Loamic, Aric) (IUSS Working Group WRB 2015). The experiment, conducted in 2004–2011, covered two crop rotation cycles: potatoes, spring barley, winter rapeseed, and winter wheat, and included the following fertilizer treatments: control (no fertilizers), NPK, manure (FYM), municipal sewage sludge composted with straw (SSCS), composted sewage sludge (SSC), dried granular sewage sludge (DGSS), “Dano” compost made from non-segregated municipal waste (CMMW), and compost made from municipal green waste (CUGW). During the field experiment, 20 t DM/ha of FYM, SSCS, SSC, DGSS, CMMW, CUGW each was applied, i.e., 10 t DM/ha per crop rotation cycle. In the years when organic fertilizers were used, supplemental nitrogen was applied to meet the crops’ requirements, subject to the total N content of fertilizers. In the other years, only mineral fertilizers were used. Selected properties of soil before setting up the field experiment are presented in Table 1.

**Table 1:** Soil characteristics before setting up the field experiment

Component		Unit	Content
C_organic_		g/kg	7.63
N_total_			0.64
C/N		–	11.92
pH 1 mol KCl/dm^3^		–	5.04
Hh		mmol(+)/kg	27.70
Available forms of heavy metals	Cu Zn Mn Pb Cd	mg/kg	1.47 10.11 104.50 9.90 0.13

The influence of manure and composts on the leaching of heavy metals from soil was evaluated in a model lysimeter experiment under controlled conditions. Soil samples were collected from field experiment, after the second crop rotation cycle with the following plant species: potatoes, spring barley, winter rapeseed, and winter wheat, from three soil horizons at a depth of 0–30, 31–60, and 61–90 cm retaining the layout of the soil profile layers. The samples were placed in lysimeter cylinders, in layers. The total amount of water applied to the lysimeter corresponded to the average annual precipitation in the Region of Warmia and Mazury, NE Poland (605 mm).

The available forms of heavy metals (Cu, Zn, Mn, Pb, Cd) in soil were determined by absorption atomic spectrometry (AAS) on the Shimadzu AA-6800 spectrophotometer after soil extraction in 1 mol HCl/dm^3^ (Karczewska and Kabała 2008). The other chemical analyses of soil were performed with the use of methods described by Ostrowska et al. (1991).

The dry matter content of organic waste materials was determined by the oven-drying (gravimetric) method (PN-EN 15934:2013-02E). Organic carbon content was determined with a spectrophotometer by the Kurmies method, after wet oxidation of organic matter with K_2_CrO_7_ in a highly acidic environment (Houba et al. 1997). Total nitrogen content was determined by the Kjeldahl method after mineralization in concentrated sulfuric(VI) acid. The concentrations of heavy metals (Cu, Zn, Mn, Pb, Cd, Cr, Ni) in FYM, SSCS, SSC, DGSS, CMMW, and CUGW were determined by AAS on the Shimadzu AA-6800 spectrophotometer, after wet mineralization in a mixture of nitric(V) acid and perchloric(VII) acid at a 4:1 ratio, with the addition of HCl. The concentrations of heavy metals (Cu, Zn, Mn, Pb, Cd, Cr, Ni) in the leachate were determined by AAS on the Shimadzu AA-6800 spectrophotometer. The determinations were completed by referring to certified material (Trace Metals - Sewage Sludge 4, Sigma-Aldrich RTC, Inc.), Table 2.

**Table 2:** The determinations referring to certified material (Trace Metals - Sewage Sludge 4, Sigma-Aldrich RTC, Inc.)

Value of determination	The content of heavy metals in Sewage Sludge 4						
	Cd	Cu	Pb	Ni	Cr	Zn	Mn
Certified value (mg/kg DM)	60.6 ± 2.96	482 ± 50.4	154 ± 12.4	163 ± 13,5	289 ± 30.4	1240 ± 181	693 ± 108
Determination value (mg/kg DM)	58.57	455.5	153.2	160.8	280.9	1075.6	615.2
Precision of determination (%)	96.7	94.5	99.5	98.7	97.2	86.7	88.8

The results of chemical analyses were analyzed statistically using Statistica 10® software. The significance of differences between means was estimated by Fischer’s test at *α* = 0.05, while the classification of percolating water and biodegradable waste materials was performed with MVPS v. 3.1 software.

## Results and Discussion

The organic waste materials used for soil fertilization differed in the content of dry matter and organic carbon and the C/N ratio. The concentrations of heavy metals in composted sewage sludge and dried granular sewage sludge were below the limits set forth in the Regulation of the Minister of Environment (2015), which allows their use in agriculture and for land reclamation for agricultural purposes (Table 3). “Dano” compost made from non-segregated municipal waste and compost made from municipal green waste were characterized by excessive lead concentrations relative to the maximum permissible Pb levels determined in the quality standards for organic and organo-mineral fertilizers, developed by the Institute of Soil Science and Plant Cultivation – National Research Institute in Puławy (Introduction...^2016^). According to Latosińska and Gawdzik (2011), the total heavy metal content of sewage sludge is not directly correlated with their release into the soil-water environment because heavy metals can exist in various forms and the proportion of mobile fractions in sewage sludge is usually low.

**Table 3:** Chemical composition of organic waste materials used in the experiment

Component		Manure (FYM)	Municipal sewage sludge composted with straw (SSCS)	Composted sewage sludge (SSC)	Dried granular sewage sludge (DGSS)	“Dano” compost made from municipal waste (CMMW)	Compost made from municipal green waste (CUGW)
DM	g/kg	222.4	586.8	403.1	851.4	746.4	788.8
C_organic_		76.20	184.7	213.9	382.7	136.2	80.20
C/N		13.1	17.2	7.3	20.3	14.6	14.1
mg/kg sm							
Cu		20.50	4.48	18.20	340.0	258.0	102.6
Zn		173.3	109.5	270.4	1310	679.5	301.0
Mn		335.4	210.6	228.0	300.5	273.6	326.8
Pb		8.40	11.46	16.54	12.40	191.9	281.6
Cd		1.61	1.42	11.08	4.58	3.10	1.70
Cr		10.20	5.80	79.50	3.00	56.70	15.00
Ni		16.98	29.30	9.90	2.09	47.48	57.00

The concentrations of selected heavy metals in the percolating water were dependent on their amounts supplied to the soil with organic compounds (Table 4). Such dependences were significant in the case of cadmium, nickel, and copper (*r* = 0.73*, *r* = 0.55*, and *r* = −0.57*, respectively). In the remaining cases, the concentration of heavy metals introduced into the soil had no significant effect on their leaching.

**Table 4:** Correlations between the content of heavy metals introduced into organic waste materials and their concentrations in soil leachate

The content of heavy metals in organic waste material	The content of heavy metals in soil filtrates						
	Cd	Cu	Pb	Ni	Cr	Zn	Mn
Cd	0.73*						
Cu		−0.57*					
Pb			−0.23				
Ni				0.55*			
Cr					−0.11		
Zn						0.21	
Mn							−0.18

*r significant at *p* ≤ 0.05, *n* = 24

The content of cadmium (*r* = 0.58*) and manganese (*r* = 0.91*) in the leachate was positively correlated with the organic carbon content of organic fertilizers (Table 5). The concentrations of copper (*r* = −0.52*) and nickel (*r* = −0.76*) decreased with increasing organic C content of organic compounds introduced into the soil. The widening of the C/N ratio significantly increased manganese leaching (*r* = 0.62*).

**Table 5:** Correlations between selected properties of organic waste materials and the heavy metal content of soil leachate

Variable	Cd	Cu	Pb	Ni	Cr	Zn	Mn
C_organic_	0.58*	−0.52*	−0.20	−0.76*	0.10	0.37	0.91*
C/N	−0.24	0.05	0.29	0.00	−0.16	0.06	0.62*

*Significant at *p* ≤ 0.05, *n* = 24

**Fig. 1:**
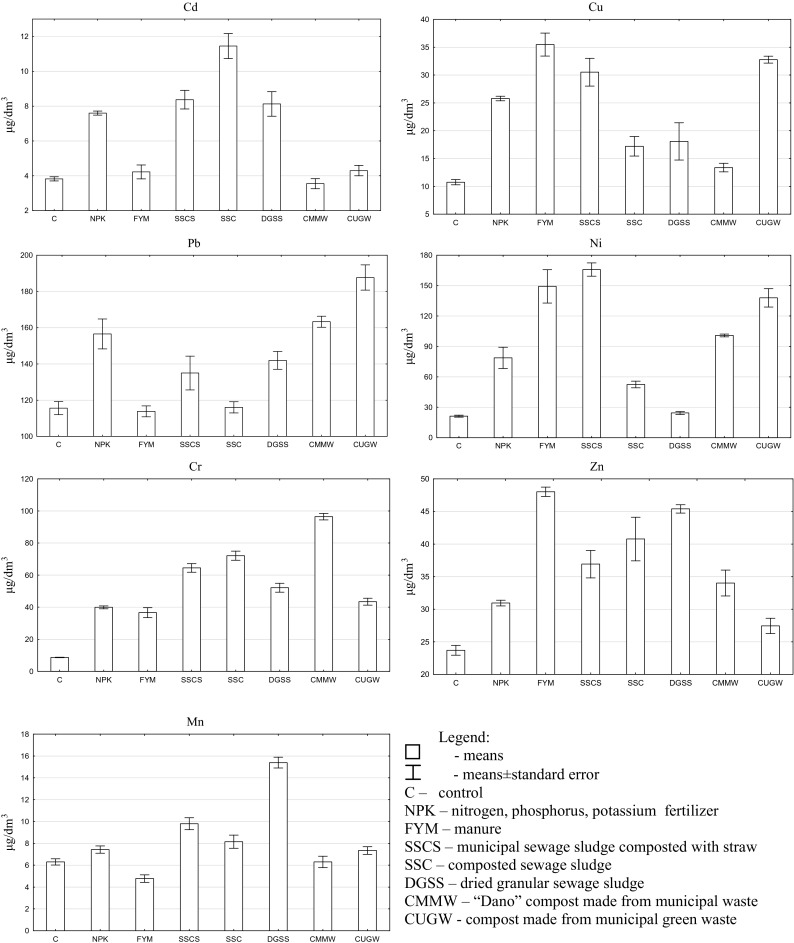
Concentrations of heavy metals in the leachate (means ± standard error)

The concentrations of heavy metals in the leachate were as follows: Cd < Mn < Cu < Zn < Cr < Ni < Pb (Fig. 1). In comparison with control soil, both mineral and organic fertilizers generally enhanced the leaching of these elements. The highest Cd concentration was noted in the leachate from soil fertilized with composted sewage sludge (11.45 μg/dm^3^). Cd concentration was significantly higher in the leachate from soil fertilized with NPK, manure, sewage sludge, and composts containing sewage sludge than in water percolating through control soil and soil fertilized with municipal compost. The leachate from control soil contained the lowest amounts of copper, nickel, chromium, and zinc (10.75, 21.25, 8.70, and 23.70 μg/dm^3^, respectively). The significantly highest concentrations of Cu and Ni were noted in the leachate from soil fertilized with manure and composts containing plant residues (sewage sludge composted with straw and compost made from municipal green waste). The greatest Cr leaching was observed in soil fertilized with compost made from non-segregated municipal waste and composted sewage sludge (96.45 and 72.12 μg/dm^3^, respectively). The greatest zinc leaching was noted in soil fertilized with manure and dried granular sewage sludge (48.03 and 45.40 μg/dm^3^, respectively). Water percolating through control soil and soil fertilized with manure had the lowest lead content (115.60 and 113.82 μg/dm^3^, respectively). The highest lead content was found in the leachate from soil fertilized with compost made from municipal green waste. Soil fertilization with dried granular sewage sludge increased manganese leaching over 2.5-fold (15.40 μg/dm^3^), compared with the control treatment. Manure-fertilized soil was characterized by the significantly lowest Mn leaching (4.77 μg/dm^3^).

This study concerned the effect of organic fertilizers on the heavy metal leaching after the second crop rotation. However, Bowszys et al. (2015) demonstrated that the zinc and copper content of soil leachate depended on crop rotation and the applied fertilizers. In both crop rotation cycles, the highest amounts of zinc (24.7 and 45.4 μg/dm^3^, respectively) were leached from soil fertilized with composted sewage sludge. In crop rotation I, the highest quantity of copper (40.7 μg/dm^3^) was also leached from soil enriched with composted sewage sludge, and in crop rotation II, with sewage sludge composted with straw (43.8 μg/dm^3^). In a study by Gondek (2010), soil fertilization with manure and sewage sludge did not significantly increase the mobility of zinc and cadmium in the first year after application. In successive years, the concentrations of mobile forms of those elements increased as a result of organic matter mineralization and progressing acidification of the soil but to a lesser degree than in plots receiving only mineral fertilizers.

The mineral content of water flowing through the soil profile may vary widely depending not only on the fertilization system but also on outflow volume and soil acidity. The concentrations of elements in the leachate from lessive soil can be also arranged in the following order: Al > Zn > Fe > Mn > Cu. Manure contributes to the leaching of zinc, iron, and manganese, and it reduces the activity and mobility of aluminum in soil. Depending on fertilization and soil acidity, 1 m^3^ of water percolating through lessive soil can leach out 29.5–251 mg Al, 53–184 mg Zn, 24.5–319 mg Fe, and 20–76.5 mg Mn per ha (Sosulski et al. 2013).

In view of the upper reference limits for trace elements set forth in the national Regulation of the Minister of Environment of 23 July 2008 on the criteria and methods for groundwater assessment, the concentrations of manganese, zinc, and copper (control, composted sewage sludge, dried granular sewage sludge, and compost made from non-segregated municipal waste) in the leachate were similar to the local hydrogeochemical background. The concentrations of the remaining metals, in particular lead, in the leachate were much higher and in some cases reached the levels characteristic of unsatisfactory and poor-quality waters. However, Brenton et al. (2007) found that, regardless of sludge dose and soil type, the concentrations of Cd, Ba, Cr, and Be in the leachate were below the maximum permissible limits for these metals in drinking water, established by the United States Environmental Protection Agency (US EPA).

Our results demonstrated also that the cadmium content of soil leachate was positively correlated (*r* = 0.39*) with the hydrolytic acidity of soil (Table 6). The concentrations of lead (*r* = −0.38*) and chromium (*r* = −0.61*) decreased with increasing values of soil Hh. Significant positive correlations were found between the levels of those metals in the leachate vs. soil pH (*r* = 0.69* and *r* = 0.74*, respectively), the organic C content of soil (*r* = 0.45* and *r* = 0.40*, respectively), and the adsorption capacity of soil (*r* = 0.36* and *r* = 0.42*, respectively). Soil pH significantly affected the nickel content of leachate (*r* = 0.39*), and the organic C content of soil influenced the manganese content of leachate (*r* = 0.48*).

**Table 6:** Correlations between selected chemical properties of soil and the heavy metal content of soil leachate

Variables	Cd	Cu	Pb	Ni	Cr	Zn	Mn
Hh	0.39*	0.11	−0.38*	−0.29	−0.61*	0.12	0.33
C_organic_	0.34	0.32	0.45*	0.19	0.40*	0.24	0.48*
pH	0.05	0.25	0.69*	0.39*	0.74*	0.18	0.17
CEC	0.34	0.20	0.36*	0.05	0.42*	0.24	0.31

*CEC* cation exchange capacity, *Hh* hydrolytic acidity

*Significant at *p* ≤ 0.05, *n* = 32

Regarding acidification, Zheng et al. (2012) demonstrated that progressive soil acidification due to acid rain increased the mobility of Cu, Pb, Cd, and Zn and their susceptibility to leaching, whereas Page et al. (2014) proved that soil pH should not be considered as the only indicator of the mobility of heavy metals, their availability to plants, or the risk of their migration to the aquatic environment. Furthermore, according to Sienkiewicz and Czarnecka (2012), an increase in the concentrations of available forms of Cu, Zn, and Mn in alkaline soils fertilized with high doses of sewage sludge (up to 280 t /ha) did not pose a threat to the environment; instead, it improved the microelement nutritional status of plants. On the other hand, the concentrations of available forms of copper, zinc, and manganese in soil regularly fertilized with manure considerably exceeded their amounts in soil receiving only mineral fertilizers (Sienkiewicz et al. 2009). Fodor and Szabó (2004) reported that heavy metals and other pollutants accumulate in the topsoil where the root mass is the highest and only a small portion of Cd, Cu, Pb, Hg, and Zn moves deeper into the soil profile. In slightly acidic soils, Cr in the form of K_2_CrO_4_ dissolves relatively easily and migrates into deeper soil layers, thus posing a threat to underground waters. In a study by McLaren et al. (2004), soil fertilization with sewage sludge reduced soil pH and increased the concentrations of Cd, Ni, and Zn in drainage leachates, but it had little effect on the concentrations of Cr, Cu, and Pb. Only approximately 1% of the metals introduced into the soil with sewage sludge were leached over a 3-year period. In only some cases, Ni and Zn concentrations in drainage leachates exceeded drinking water standards. Therefore, the leaching of those metals was unlikely to pose a major environmental threat. The dissolved organic carbon contained in sewage sludge and applied to calcareous soil may facilitate Ni leaching due to the formation of soluble Ni-organic complexes (Mamindy-Pajany et al. 2013).

**Fig. 2:**
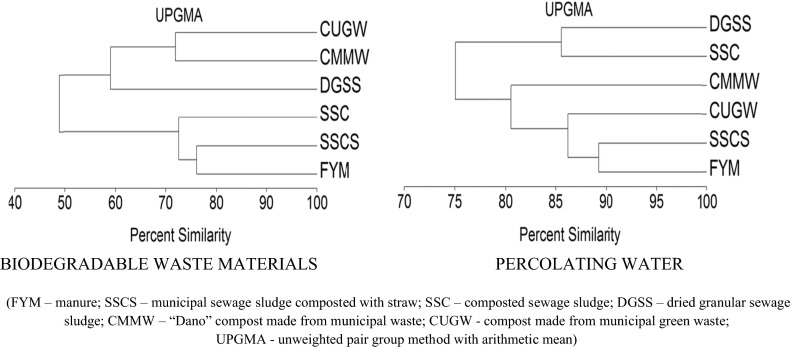
A hierarchical classification of heavy metal content in biodegradable waste materials and percolating water

A cluster analysis of the content of heavy metals in biodegradable waste materials and percolating water based on “percent similarity” demonstrated considerable differences (Fig. 2). In both cases, two objects, i.e., manure (FYM) and municipal sewage sludge composted with straw (SSCS) composed the first group with the highest similarity. The most similar content of heavy metals in percolating water was also between dried granular sewage sludge (DGSS) and composted sewage sludge (SSC). They created the second group which was completely different compared with the biodegradable waste materials used. Analyzing the similarity of the samples with biodegradable waste materials, the compost made from municipal green waste (CUGW) and “Dano” compost made from municipal waste (CMMW) were very similar and formed a group with DGSS, whereas in percolating water, they were more similar to SSCS and FYM. Thus, the origin of the waste materials used in the experiment decided about the groundwater quality. The processed municipal waste (DGSS and SSC) changed the whole similarity arrangement, and they caused the highly similar contamination in percolating water.

Comparing with the other studies, the divergence in the degree of heavy metals leaching may have different reasons. Fertilizers applied at rates exceeding the nutrient requirements of plants may alter the ionic equilibrium of the soil solution and nutrient transport to groundwater (Gondek 2009). Single applications of sewage sludge, even at high doses, do not significantly increase heavy metal leaching from soil, in comparison with manure and mineral NPK fertilization. However, due to the positive balance of those elements in soil, long-term application of sewage sludge could become a problem, especially that high soil acidity increases the mobility of heavy metals and their susceptibility to leaching (Sevel et al. 2014). Milinovic et al. (2014) claim that sludge drying before application to soil generally decreases the leaching of Zn, Pb, and Cr and increases the leaching of Cu and Ni. Faridullah et al. (2012) observed greater leaching of Mn, Cu, Ni, and Zn from soil fertilized with poultry litter, compared with control soil. According to Riedel et al. (2015), the addition of biochar to soil contaminated with heavy metals may considerably increase their sorption; thus, it may reduce the risk of groundwater contamination.

## Conclusions

1. The concentrations (μg/dm^3^) of heavy metals in the leachate were as follows: Cd (3.6–11.5) < Mn (4.8–15.4) < Cu (13.4–35.5) < Zn (27.5–48.0) < Cr (36.7–96.5) < Ni (24.4–165.8) < Pb (113.8–187.7).
2. Soil fertilization with organic waste materials did not contaminate the percolating water with manganese or zinc, whereas the concentrations of Cd, Ni, and Pb increased to the levels characteristic of unsatisfactory and poor-quality waters.
3. The copper and nickel content of percolating water depended on the concentration of those metals introduced into the soil with organic waste materials.
4. The concentrations of Cd in the leachate increased, whereas the concentrations of Cu and Ni decreased with increasing organic C content of organic fertilizers. The widening of the C/N ratio contributed to Mn leaching.
5. The concentrations of Pb, Cr, and Mn in the percolating water were positively correlated with the organic C content of soil. The concentrations of Pb and Cr were also dependent on the hydrolytic acidity, pH, and adsorption capacity of soil (CEC).
6. The agricultural use of soils can cause an increased leaching of heavy metals into groundwater.
